# Emergence of clade 3 emm89 group A Streptococcus in Queensland, Australia

**DOI:** 10.1099/mic.0.001611

**Published:** 2025-09-25

**Authors:** Rikki Marie Ann Graham, Ning-Xia Fang, Susan M. Moss, Cameron Moffatt, Amy V. Jennison

**Affiliations:** 1 Public Health Microbiology and Queensland Public Health and Infectious Diseases Reference Genomics (Q-PHIRE Genomics), Public and Environmental Health Reference Laboratories, Queensland Health, Brisbane, Australia; 2 OzFoodNet, Public Health Intelligence Branch, Queensland Public Health and Scientific Services, Queensland Health, Brisbane, Australia

**Keywords:** emm89, genomic epidemiology, *Streptococcus pyogenes*, whole-genome sequencing

## Abstract

The emergence of a new clade of *emm*89 group A *Streptococcus* (GAS) (clade 3) has been described in several countries. Strains in this clade have been reported to have genomic characteristics that lead to increased expression of virulence factors and may confer a selective advantage over previous *emm*89 strains. To investigate whether clade 3 GAS is present in the *emm*89 GAS population of Queensland, Australia, all *emm*89 GAS isolates received by the Queensland Public Health Microbiology Reference Laboratory since *emm* typing began in the early 2000s underwent genomic sequencing and analysis. Analysis of sequences from 293 *emm*89 GAS isolates demonstrated the presence of distinct genomic groups in the Queensland *emm*89 GAS population. Unlike *emm*89 GAS populations described in the UK and USA, which were mostly ST101 and ST407, there were a relatively high number of ST142 and ST812 strains in the Queensland *emm*89 GAS population. However, the majority of Queensland isolates belonged to clade 3, with 80% (*n*=233) of *emm*89 GAS isolated from 2006 onwards belonging to this clade. All Queensland clade 3 isolates had the reported genomic features associated with higher virulence potential including increased streptolysin O production and an acapsular phenotype. Clade 3, which has emerged to become the dominant clade of *emm*89 GAS in Europe and the USA, is now also the dominant clade in Queensland.

## Data Summary

Sequence files for the isolates described in this study have been uploaded to the SRA archive, with project accession number PRJEB76673. A full list of sequences with associated accession numbers can be found in Table S1 (available in the online Supplementary Material).

## Introduction

*Streptococcus pyogenes* [group A *Streptococcus* (GAS)] causes a range of human infections from relatively mild conditions such as pharyngitis or impetigo to invasive infections including septicaemia and necrotizing fasciitis, as well as serious post-infectious sequelae such as acute rheumatic fever, acute poststreptococcal glomerulonephritis and rheumatic heart disease [1,2]. Factors that contribute to invasive GAS (iGAS) infection risk can include First Nation’s status, extremities of age (>75 years or <5 years) and being a close contact of a case in either a household or institutional setting, including residential aged care facilities (RACFs) [3].

In the Australian state of Queensland (QLD), iGAS disease has been notifiable under the public health act since December 2005 [4]. The incidence of iGAS in QLD between 2006 and 2015 was 4.5 notifications per 100,000 people per year, with notification rates in Indigenous Australians eight times higher than in non-Indigenous people [3]. This increased incidence of GAS disease in Indigenous Australians is more pronounced in remote regions such as North QLD (NQ), which is sparsely populated and geographically remote compared to the rest of QLD [5]. Residents of aged care facilities are also at a higher risk of iGAS infection and have a higher case fatality rate compared with the broader public [6,7].

GAS are classified into more than 240 *emm* types based on the sequence of the gene encoding the serotype-defining M protein (*emm* gene) [8]. In the past decade, *emm*89, which is the second most common *emm* type in QLD, has been reported as an increasing cause of disease in many countries worldwide, alongside reports of a new clade of *emm*89 emerging [9,16]. Genomic analysis of *emm*89 GAS has identified that it exists in three major genetically distinct clades designated as clades 1, 2 and 3. Clade 3, the most recent clade to emerge, began emerging in the 1990s but by the mid-2000s had displaced clades 1 and 2 to become the dominant clade of *emm*89 GAS in Europe and the USA [12,14, 16]. There is a high degree of genomic difference between the clades, mainly due to horizontal transfer of genomic elements including virulence factors. Major differences in clade 3 strains include a variant in the promoter region of the *nga-ifs-slo* locus that leads to an increase in streptolysin production and the replacement of the hyaluronic acid capsule synthesis locus with a 157 bp region, leading to an acapsular phenotype [14,16, 17].

A similar phenomenon has occurred in *emm*1 GAS, with a new variant reported in the UK associated with an increase in invasive infection. This new variant, named M1_UK_, also expanded and replaced the previous clone of M1 GAS in the UK [18,19]. Investigations into QLD *emm*1 GAS found that from 2016, M1_UK_ has also become the dominant *emm*1 variant in QLD [20].

The spread of clade 3 *emm*89 GAS globally, leading to it becoming the dominant clone in several countries, and the known expansion in QLD of the global *emm*1 strain M1_UK_, would suggest that an expansion of clade 3 *emm*89 GAS in QLD is likely to have occurred. However, until now, this has not been investigated.

The goal of our study was to investigate the presence of clade 3 *emm*89 GAS in QLD by analysing the genomic sequences of all *emm*89 GAS isolates submitted to the QLD Public Health Microbiology Reference Laboratory between 2002 and 2022. We found that clade 3 *emm*89 GAS emerged in QLD prior to 2006 and has rapidly replaced clade 2 to become the dominant clone in QLD.

## Methods

### Bacterial strains

The Public Health Microbiology (PHM) laboratory is the state reference laboratory for the state of QLD and receives all iGAS isolated in the state. All *emm*89 GAS isolates received by the PHM laboratory between 2002 and 2022 (*n*=293) were included in this study (Table S1). The isolates include all invasive *emm*89 GAS notified in QLD during this time as well as any non-invasive isolates that were sent to the laboratory for typing. Invasive isolates were defined as isolates from normally sterile sites, as well as necrotizing fasciitis isolates.

### Whole-genome sequencing

GAS isolates were cultured on sheep’s blood agar at 37 °C with 5% CO_2_. DNA was extracted from cultures using the QiaSymphony DSP DNA Mini kit (Qiagen, Germany) according to the manufacturer’s instructions. Libraries were prepared with a Nextera XT DNA sample preparation kit (Illumina, USA) and sequenced on the NextSeq500 with the 500 Mid Output v2.5 kit (300 cycles) (Illumina) according to the manufacturer’s instructions.

### Analysis

Sequences were trimmed using Trimmomatic (v0.36) [21] and assembled using SPAdes (v3.13.1) [22]. SNP analysis used to identify relationships between isolates and clades was carried out using Snippy (v4.3.6) with the clinical strain H293 (accession: HG316453) as a reference. Gubbins (v2.4.1) [23] was used to identify regions of recombination which were excluded from phylogenetic analysis. Maximum likelihood trees were generated using FastTree (v2.1.11) [24] and visualized using iTol (v6). Genomic assemblies were screened for the presence of virulence genes using Abricate (v1.0.1) and the virulence factor database (vfdb). Ridom Seqsphere+ (v9.0.8) was used to identify MLST sequence types (STs) using the scheme hosted at pubMLST [25]. Ridom Seqsphere+ (v9.0.8) was also used to identify *nga* promoter variants and *nga* alleles. Case rates were calculated per 100,000 persons, based on the 2016 QLD population. For the purposes of this study, NQ includes the Cairns and Hinterland, North West, Torres and Cape and Townsville hospital and health services (HHS) regions, Central QLD (CQ) includes the CQ, Central West and Mackay HHS regions and South QLD (SQ) includes the Darling Downs, Gold Coast, Metro North, Metro South, South West, Sunshine Coast, West Moreton and Wide Bay HHS regions.

## Results

### *emm*89 GAS in QLD

**Fig. 1.**
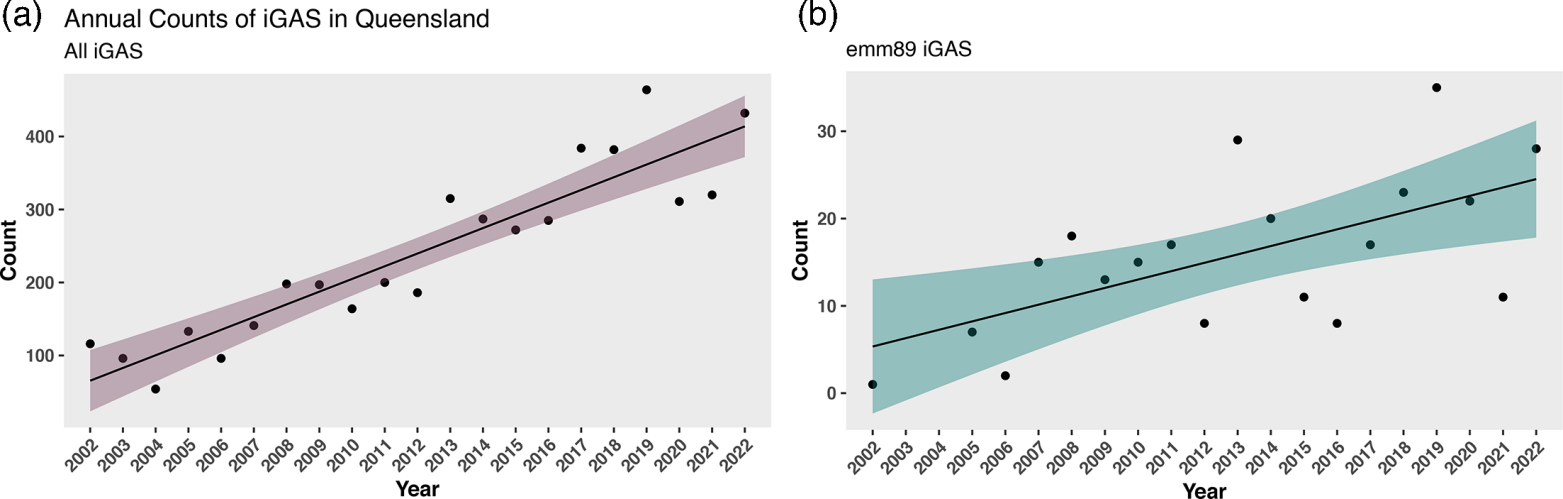
Annual counts of iGAS (**a**) and *emm*89 iGAS (**b**) isolates received by the QLD PHM laboratory. Black dots indicate absolute counts, the linear trend is shown in black and the shaded areas indicate the standard error.

On average, the incidence of invasive disease caused by *emm*89 GAS has been steadily increasing since 2002, as has the incidence of iGAS in general (Fig. 1), and *emm*89 has consistently remained in the top five *emm* types seen in QLD since 2006. Between 2002 and 2022, there were 293 *emm*89 GAS isolates in total submitted to the PHM laboratory, and 226 of these were invasive isolates (iGAS). The age distribution of cases was from 2 months to 100 years of age, with a median age of 52 and a ratio of males to females of 52%–48%, respectively.

### *emm*89 subtypes and STs

Two subtypes of *emm*89 were observed amongst QLD *emm*89 GAS: *emm*89.0 which made up the majority of isolates (*n*=272) and *emm*89.14 (*n*=21). MLST analysis indicated that there were nine different MLST STs amongst QLD *emm*89 GAS: ST101 (*n*=227), ST142 (*n*=36), ST646 (*n*=5), ST812 (*n*=20), ST912 (*n*=1), ST1035 (*n*=1), ST1090 (*n*=1), ST1095 (*n*=1) and ST1446 (*n*=1). There was an association between *emm*89 subtype and ST, with all ST812 and ST1035 having the *emm*89.14 subtype and all other STs having the *emm*89.0 subtype.

**Fig. 2.**
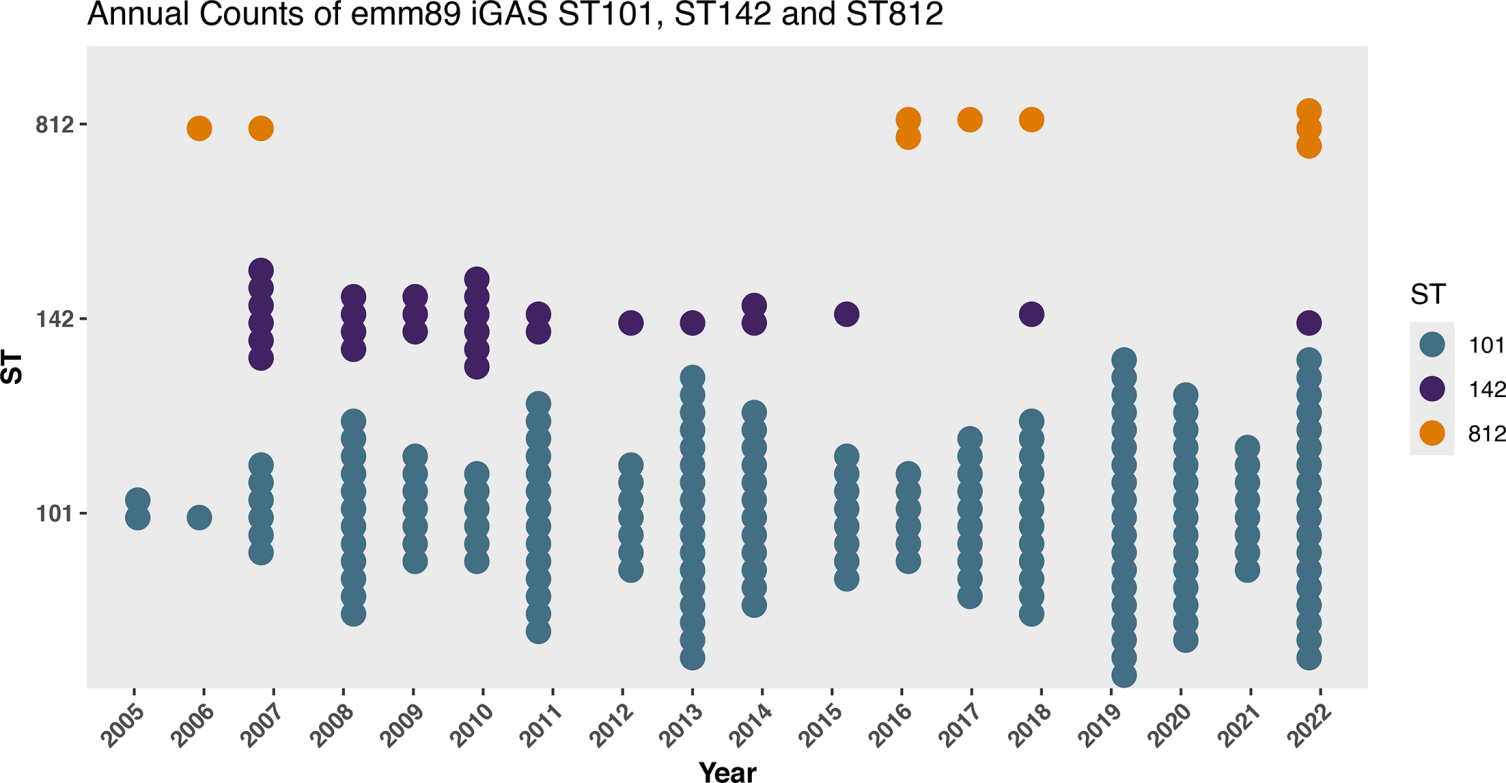
Annual counts of ST101, ST142 and ST812 *emm*89 isolates received by the QLD PHM laboratory. Each dot represents one isolate.

The relative numbers of each ST amongst QLD *emm*89 iGAS have changed over time. Prior to 2011, the number of ST142 isolates was comparable to the number of ST101 isolates. However, since 2011, the proportion of ST142 *emm*89 GAS has fallen with a concurrent increase in ST101, which has since become the dominant ST (Fig. 2). In this comparison, only iGAS isolates were considered as not all non-invasive isolates are sent to the PHM laboratory for typing.

### Region

**Fig. 3.**
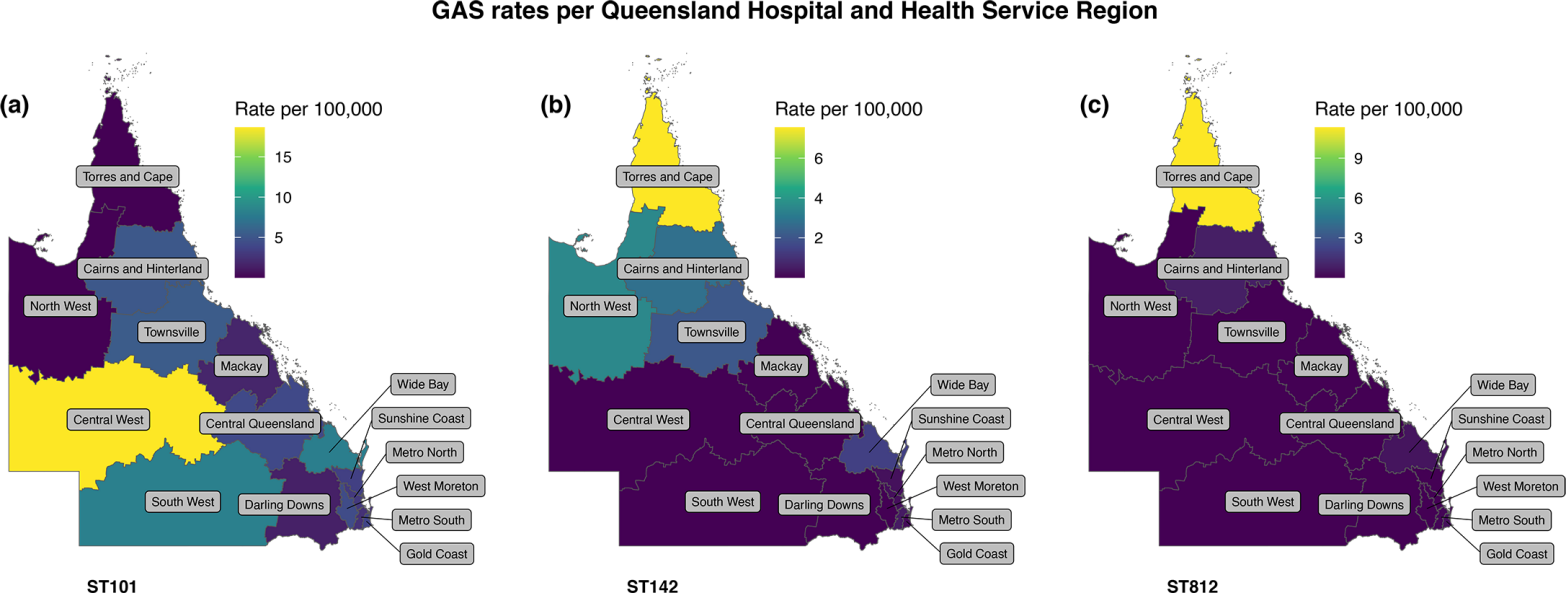
Maps of QLD showing the different hospital and health service regions with the rates of iGAS per 100,000 persons. (a) ST101, (b) ST142 and (c) ST812.

The majority of *emm*89 GAS were isolated from the SQ region (65%, *n*=190), followed by NQ (25%, *n*=72) and CQ (5%, *n*=16) with the remainder isolated from interstate or overseas travellers (5%, *n*=15). When the rate of *emm*89 iGAS per 100,000 persons was investigated, the rate in NQ was more than double that of SQ (9.1 cases per 100,000 vs 3.8 cases per 100,000). In particular, we found that the rates of ST142 and ST812 were nine times higher in NQ than in SQ (ST142 : 2.7 cases per 100,000 vs 0.3 cases per 100,000 and ST812 : 0.9 cases per 100,000 vs 0.1 cases per 100,000) (Fig. 3).

### Phylogeny of *emm*89 gas in QLD

**Fig. 4.**
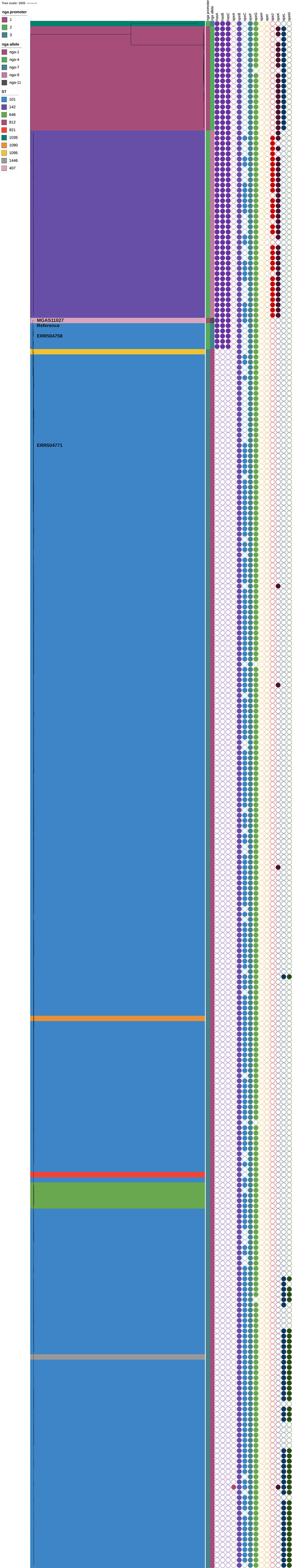
Maximum likelihood tree of 293 QLD *emm*89 GAS and representative sequences from clades 1, 2 and 3 (MGAS11027, ERR504758 and ERR504771, respectively, indicated on the tree), built using 21376 SNP differences relative to the reference sequence H293. Clades are shaded according to ST, and *nga* promoter type and *nga* allele variant are indicated by the coloured strip shown to the right of the tree. Presence or absence of virulence factors is indicated by the circles to the right of the tree. Filled circles represent the presence of the gene, and empty circles represent the absence of the gene. Circles are coloured to aid in visualization. Branch length represents genetic distance as indicated by the scale bar.

**Fig. 5.**
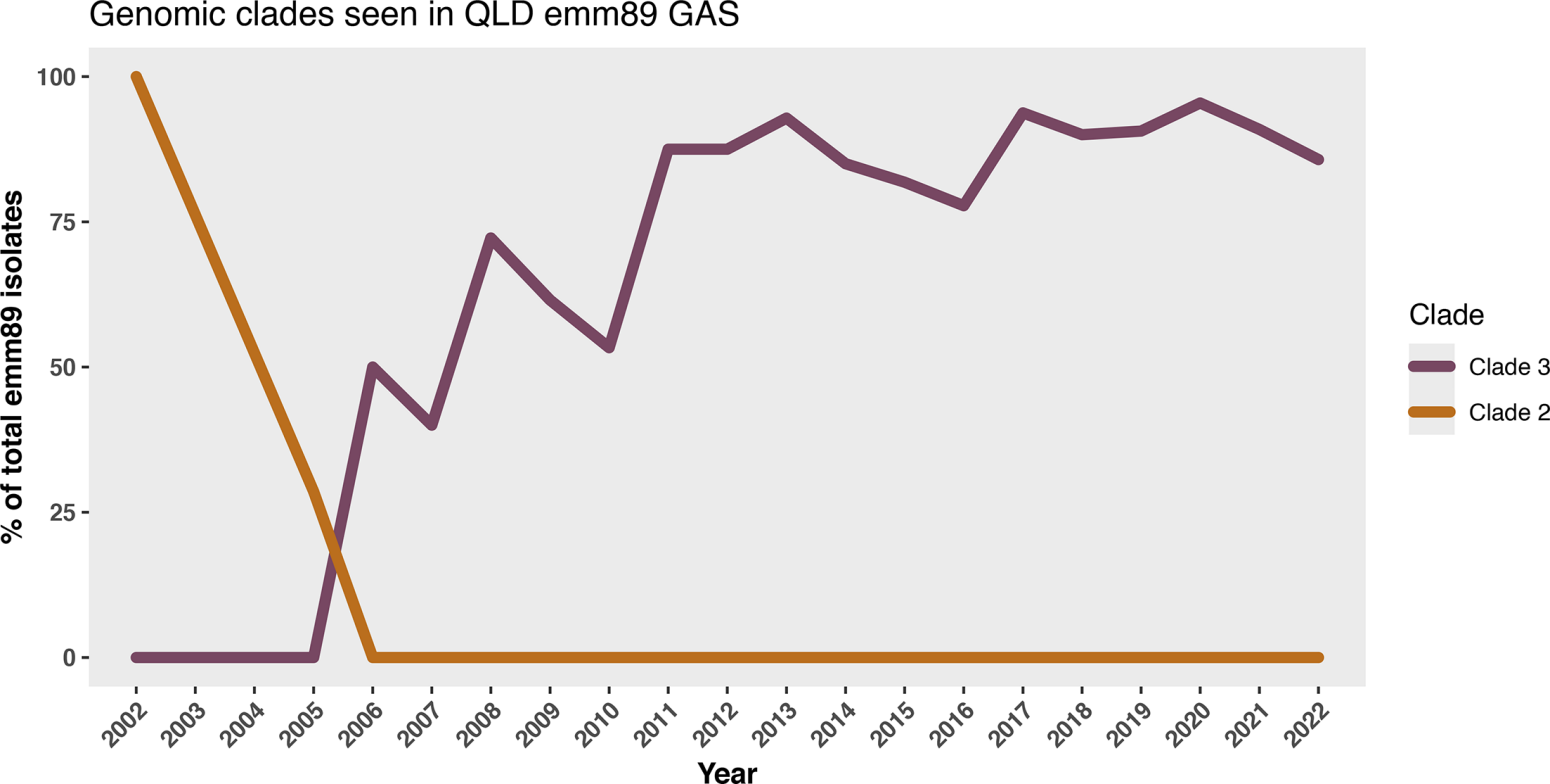
Frequency of *emm*89 isolates belonging to clades 2 and 3 received each year by the QLD PHM laboratory.

Core SNP analysis of the 293 *emm*89 GAS received since 2002, in the context of representative sequences from clade 1 (MGAS11027), clade 2 (ERR504758) and clade 3 (ERR504771), showed that QLD isolates clustered into four groups largely based on ST. The largest group, named the ST101 group, was made up of ST101 (*n*=227) and its single locus variants (SLVs) ST646 (*n*=5), ST921 (*n*=1), ST1090 (*n*=1), ST1095 (*n*=1) and ST1446 (*n*=1). Both the clade 2 and clade 3 sequences belonged to this group. The three additional groups seen in QLD isolates consisted of ST142 (*n*=36), ST812 (*n*=20) and a single ST1035. None of the QLD isolates belonged to clade 1, and the number of QLD isolates belonging to clade 2 was low, with only three isolated from 2002 and 2005 belonging to this clade (Fig. 4). None of the *emm*89 GAS isolated after 2005 belonged to clade 2. So, by 2006, clade 3 was already dominant amongst QLD *emm*89 (Fig. 5). Numbers of ST142 have also been declining over time, while numbers of clade 3 have been increasing. There was a high level of genomic difference seen between the different QLD ST groups, with ~400 SNPs present between isolates from the ST101 group and ST142 group and ~5,000 SNPs present between the ST101 group and the ST812 and ST1035 groups. This is higher than the distance seen between clade 1 and clades 2 and 3, which in our analysis was ~100 SNPs.

### ST101 group

**Fig. 6.**
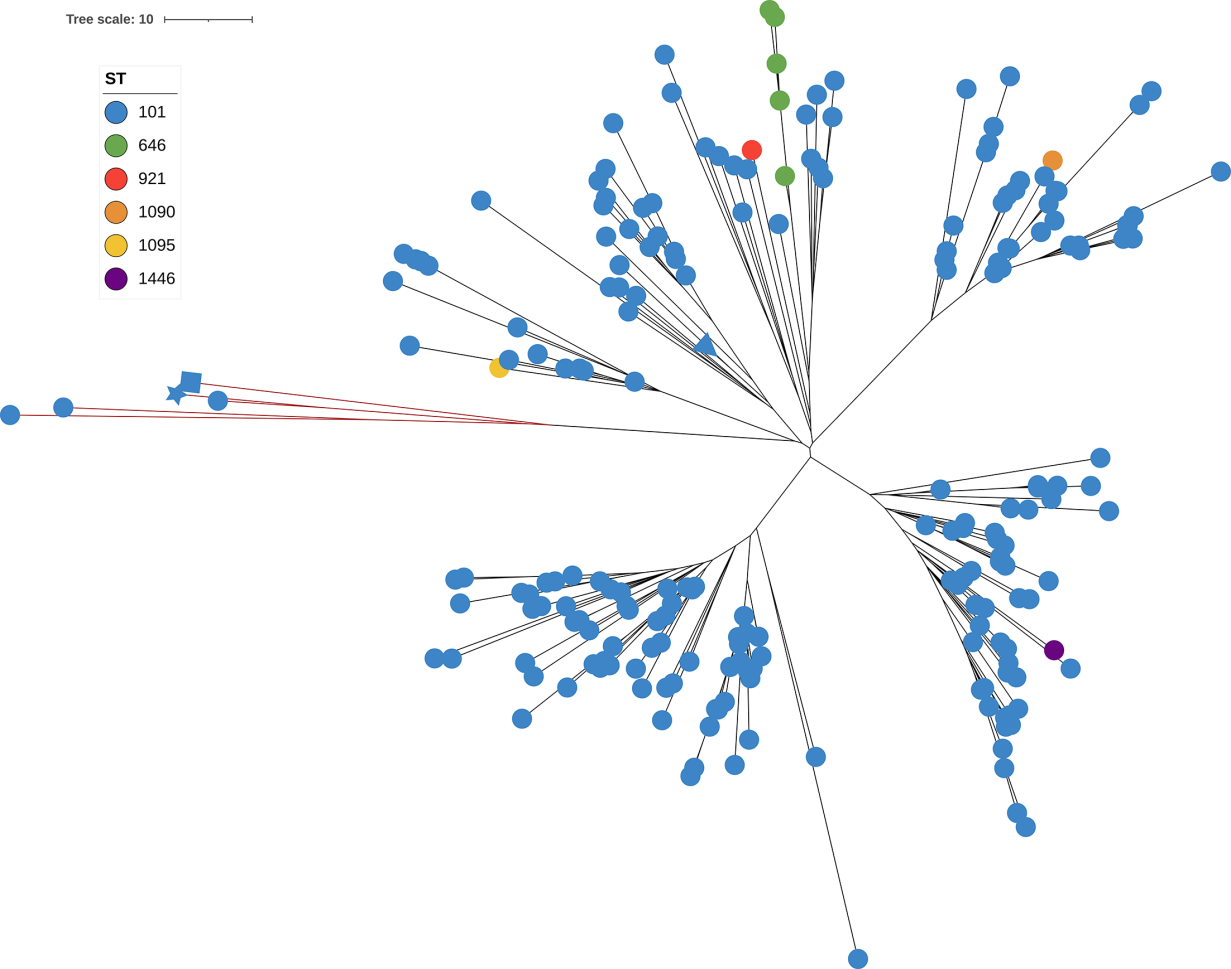
Maximum likelihood tree of 236 QLD *emm*89 GAS (circle symbols) and representative sequences from clade 2 (ERR504758, star symbol) and clade 3 (ERR504771, triangle symbol), built using 1985 SNP differences relative to the reference sequence H293 (square symbol). Red branches indicate clade 2 and black branches indicate clade 3. The colour of symbols indicates ST as shown in the legend. Branch length represents genetic distance as represented by the scale bar.

The majority of QLD *emm*89 GAS isolates belonged to the ST101 group (81%, *n*=236). As this is the largest group amongst QLD *emm*89 GAS and clade 3 is also made up of ST101 strains, the ST101 group was further analysed independently of the ST142, ST812 and ST1035 groups to improve the resolution of the phylogeny. A total of 236 ST101 group isolates belonged to clade 3. Although in international reports this clade consisted only of ST101, the QLD isolates falling into this clade also included the SLVs ST646, ST921, ST1090, ST1095 and ST1446. The SLV isolates did not cluster independently of the ST101 strains, and although the ST646 did all cluster together, they were not highly divergent from the rest of the clade 3 (Fig. 6).

### Invasive vs non-invasive disease

**Fig. 7.**
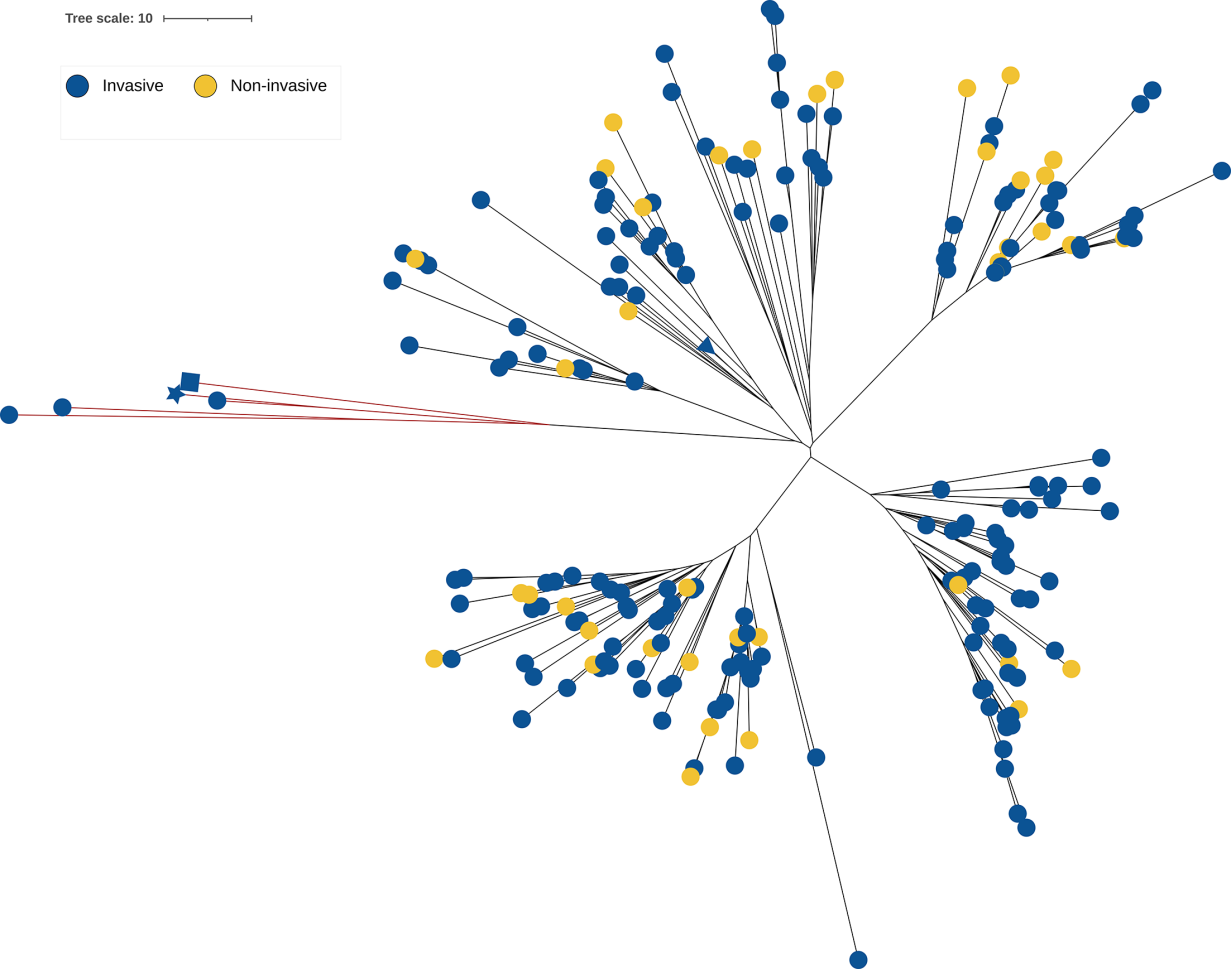
Maximum likelihood tree of 236 QLD *emm*89 GAS (circle symbols) and representative sequences from clade 2 (ERR504758, star symbol) and clade 3 (ERR504771, triangle symbol), built using 1985 SNP differences relative to the reference sequence H293 (square symbol). Red branches indicate clade 2 and black branches indicate clade 3. The colour of symbols indicates the disease type of the isolate as shown in the legend. Branch length represents genetic distance as indicated by the scale bar.

Of the 293 *emm*89 received between 2002 and 2022, 226 were invasive and 67 were non-invasive. No association was seen between *emm*89 subtype or ST and invasiveness. Phylogenetic analysis of ST101 isolates found that invasive and non-invasive isolates did not cluster into separate groups but were interspersed throughout the phylogenetic tree (Fig. 7).

### Virulence genes

The presence or absence of a range of virulence genes was assessed and is shown in Fig. 4. Several virulence genes were either present or absent in all isolates or did not show any association with any particular groups. However, there were some virulence genes that showed an association with one or other group(s), as described below.

#### *hasABC* operon

The *hasABC* operon required for the expression of the hyaluronic capsule was absent in all clade 3 isolates and was replaced by a short 157 bp region. The ST142, ST812 and clade 2 isolates all possessed the full-length *hasABC* locus (Fig. 4).

#### *nga-ifs-slo* operon

QLD *emm*89 GAS belonging to clade 3 had the variant 3 *nga-ifs-slo* operon promoter and *nga* allele 1 described in [14], consistent with what has been reported in international clade 3 isolates. Clade 2, ST142 and ST1035 isolates all had the variant 2 promoter, but clade 2 and ST1035 both had *nga* allele 7 while ST142 had *nga* allele 9. ST812 isolates had the variant 1 *nga-ifs-slo* promoter with *nga* allele 4 (Fig. 4).

#### Other virulence genes

Analysis of the genomes for genes for *speA*, *speB*, *speC*, *speF*, *speG*, *speH*, *speI*, *speJ*, *speK*, *speL* and *speM* found that the *speH* and *speI* genes were absent from all isolates, and the *speA* gene was absent from all but one isolate. The *speB* and *speF* genes were present in all isolates, and *speG* was present in 99% of isolates (289/293). The *speJ* gene was present only in ST142 isolates, and *speK* was present in 33/36 ST142 and 18/20 ST812 but only in 3/233 clade 3 isolates. The *speL* gene was absent from ST142 group isolates but present in all ST812 isolates. Neither ST142 nor ST812 group isolates had *speM*, while 44/233 clade 3 isolates did have this gene. The *speC* gene was present in 80% of clade 3 isolates (186/233) and 50% of ST142 isolates (18/36) and was absent in all clade 2 and ST812 isolates (Figs4  8).

**Fig. 8.**
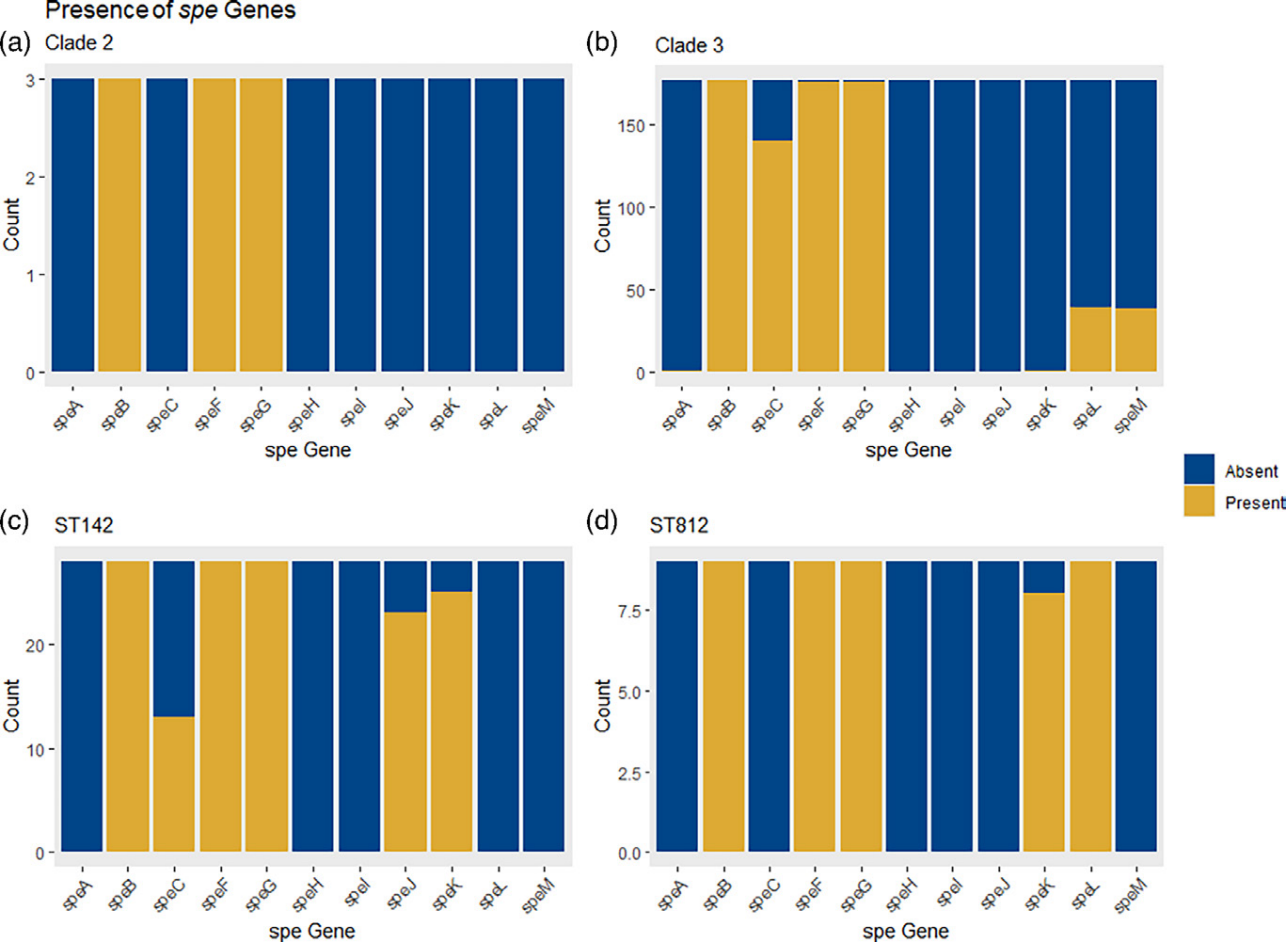
Presence or absence of *spe* genes in clade 2 (**a**), clade 3 (**b**), ST142 (**c**) and ST812 (**d**) *emm*89 isolates received by the QLD PHM laboratory.

### GAS *emm*89 in QLD RACFs

**Fig. 9.**
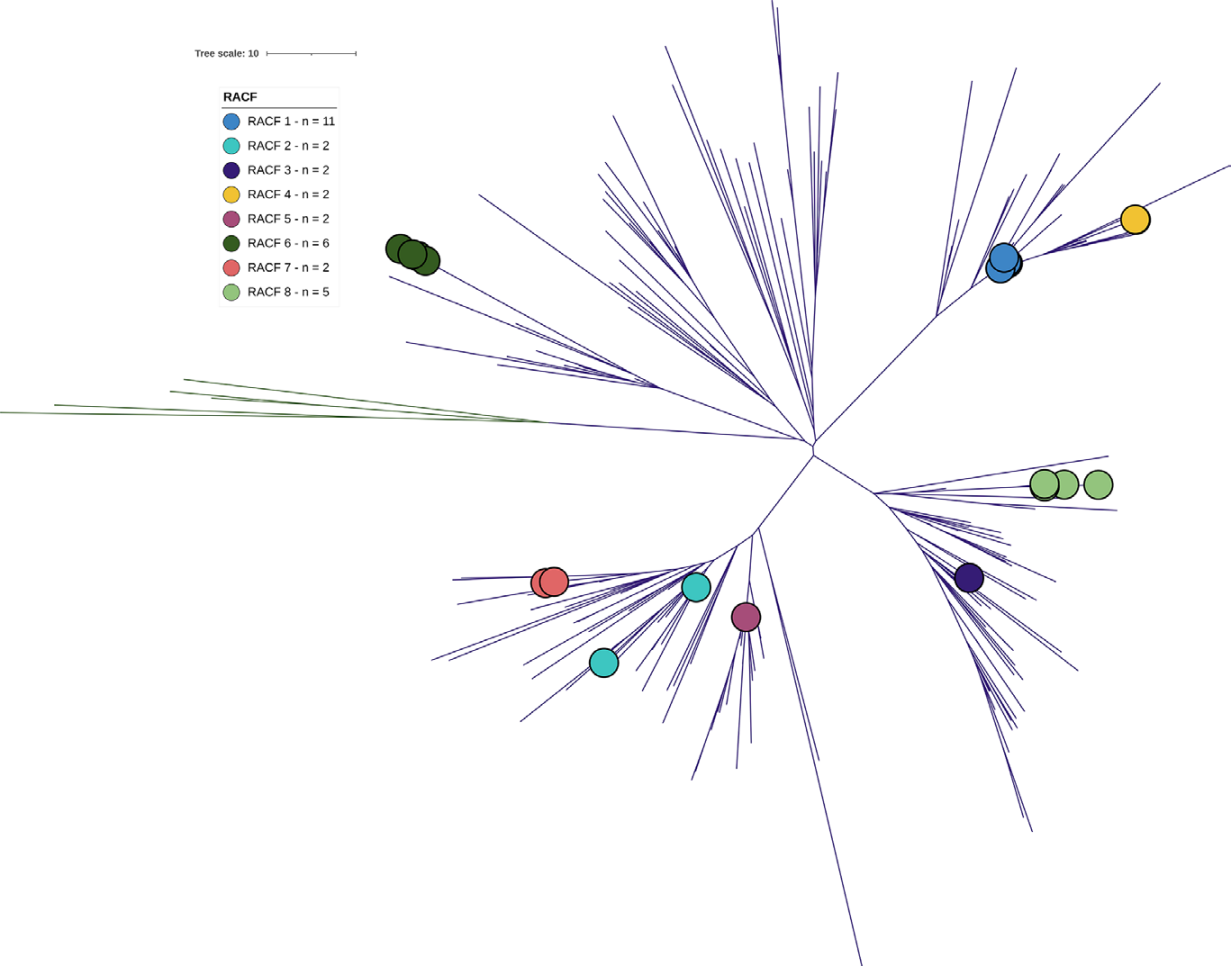
Maximum likelihood tree of 236 QLD *emm*89 GAS, built using 1,985 SNP differences relative to the reference sequence H293 (square symbol). Green branches indicate clade 2 and purple branches indicate clade 3. The colour of symbols indicates RACF as shown in the legend. Branch length represents genetic distance as indicated by the scale bar.

Between 2013 and 2022, eight clusters of GAS *emm*89 were identified in RACFs. Sequence analysis of these isolates showed they all belonged to clade 3 but that there was a high level of genetic distance between individual RACF clusters. With one exception, isolates from the same RACF were genetically tightly clustered (Fig. 9). Isolates from two cases thought to be a potential cluster (RACF2) were not closely related genetically to each other and so were found not to be a cluster. These two isolates were collected 3 years apart. No particular virulence factors were found to be associated with the RACF isolates; however, all did have *speC* except for the two from RACF2.

## Discussion

Over the past decade, reports have shown that a newly emergent clade of *emm*89 GAS has spread globally, replacing previous *emm*89 GAS clades [10,16]. In this study, we have shown that since 2006, this emergent clade, clade 3, has also been the dominant strain of *emm*89 GAS in QLD. QLD clade 3 strains shared the same major characteristics as those reported internationally. They all had a deletion of the *hasABC* locus involved in the synthesis of the hyaluronic acid capsule, and they all possessed the variant 3 version of the *nga-ifs-slo* operon promoter with *nga* allele 1. It has been shown that the hyaluronic acid capsule is an important virulence factor and that GAS that do not produce hyaluronic acid are associated with milder disease [26]. However, although clade 3 *emm*89 GAS demonstrate an acapsular phenotype, this may be compensated for by the increased expression of NADase and streptolysin O caused by variants in the promoter region of *nga-ifs-slo* that affect the transcription of the genes for both *nga* and *slo* and thus the expression of NADase and streptolysin O. Promoter variant 3 of *nga-ifs-slo* is associated with epidemic strains of M1 GAS and clade 3 *emm*89 GAS from the UK and USA [16,17]. QLD isolates from the ST142 and ST812 groups sit outside of clades 1, 2 and 3, with ~400 and 5,000 SNPs, respectively, between isolates in these groups and those in clades 1, 2 and 3. Isolates from ST142 and ST812 had *nga-ifs-slo* promoter variants 1 and 2, respectively. In a study of streptolysin O production and mouse survival, isolates with *nga-ifs-slo* promoter variants 1 and 2 produced less streptolysin O and were associated with a higher mouse survival rate than isolates with *nga-ifs-slo* promoter variant 3 [14], so it is likely that ST142 and ST812 isolates have reduced streptolysin O production compared with clade 3 isolates. ST142 and ST812 isolates did however carry a different array of virulence genes compared with clade 3 isolates. Further experimental studies would be useful to confirm whether or not these differences in virulence factor genes have an impact on the virulence of ST142 and ST812 strains.

Only three clade 2 isolates were seen amongst the QLD *emm*89 GAS, and these were collected in 2002 and 2005. However, it should be noted that iGAS disease only became notifiable in QLD in December 2005, and prior to this, not all iGAS were being sent to the QLD PHM laboratory. It is therefore possible that the incidence of clade 2 *emm*89 GAS was higher prior to 2005, but this is not reflected in the isolates received by the laboratory and included in this study [3]. It should also be noted that counts of *emm*89 GAS received by the laboratory before 2006 were low and may not have been an accurate representation of true *emm*89 GAS diversity in QLD. For this reason, analysis of GAS rates in QLD only included isolates collected in 2006 and later. In addition, only iGAS were included in this analysis as not all non-invasive isolates are sent to the PHM laboratory for typing.

In our analysis, we did not find any particular clone amongst QLD *emm*89 GAS that was associated with invasive vs non-invasive disease, although an exhaustive study of non-invasive disease isolates was not performed. Instead, we found that invasive and non-invasive isolates were interspersed throughout the SNP phylogeny. This is consistent with previous reports of both *emm*89 and GAS in general where genetic differences between invasive and non-invasive isolates were found to be minimal [9,27,29]. Similarly, although all *emm*89 GAS associated with clusters in RACF belonged to clade 3, we did not find any specific clone that was associated with RACF isolates.

What we did find, however, was that there appears to be a high level of diversity present in *emm*89 GAS from QLD and that although clade 3 is the dominant clade in QLD, the highly divergent strains ST142 and ST812 continue to persist in the community. *emm*89 GAS has previously been reported to have a higher level of genetic diversity than other *emm* types [9], and studies in the USA and Canada have identified distinct clades that are genetically distant to clades 1, 2 and 3 [5,30].

Interestingly, a recent study from Japan identified that a large proportion of their clade 3 isolates are ST646, an SLV of ST101 [31,32]. We also found five ST646 isolates in the QLD *emm*89 population, collected between 2016 and 2022 although they have remained in low numbers relative to ST101.

We found that ST142 and ST812 strains are associated with the NQ region, which is geographically remote and sparsely populated. The population of NQ has a large proportion of Indigenous Australians who are uniquely vulnerable to GAS and other infectious diseases for a variety of reasons including social disadvantage and inadequate housing conditions [33,36], and the geographic isolation of this region has led to the emergence of distinct populations of GAS as well as other infectious diseases [37,38]. The information regarding indigenous status available to the laboratory is incomplete however, and so any association between these STs and this population group could not be further explored. ST142 and ST812 have not been widely reported internationally, and the *S. pyogenes* pubMLST database [25] (accessed 11 April 2025) has one record of a non-Australian ST142 from Germany and a single ST812 from Norway. Two ST142 and two ST812 were reported from the USA in a study of *emm*89 GAS from the USA, Finland and Iceland, where they were described as outliers from the majority of *emm*89 isolates analysed in their study [9]. However, a recent study of GAS in New Zealand identified ST142 and ST812 amongst the *emm*89 GAS collected from Auckland children. These were present in numbers relative to ST101 strains that were similar to that seen in QLD, highlighting a similarity between the *emm*89 GAS populations of both countries [39].

## Conclusions

GAS *emm*89 clade 3, which has emerged in several countries to become the dominant clade, has also become the dominant clade amongst *emm*89 GAS in QLD. It is likely that clade 3 is also the dominant clade in the rest of Australia; however, unlike in QLD where iGAS has been notifiable since 2006, iGAS only became nationally notifiable in 2021, so there is limited information on the genomic epidemiology of GAS in other states. Nevertheless, genomic analysis of *emm*89 from other states would be beneficial in providing more information on the prevalence of clade 3 throughout the country. Such an analysis would also be useful in determining the prevalence of ST142 and ST812 in other regions of Australia to elucidate whether these STs are found throughout Australia or are specific to QLD.

## Supplementary material

10.1099/mic.0.001611 Uncited Table S1.

## Abbreviations

CQ: Central Queensland
GAS: group A *Streptococcus*
HHS: hospital and health services
iGAS: invasive group A *Streptococcus*
NQ: North Queensland
PHM: Public Health Microbiology
QLD: Queensland
RACF: residential aged care facility
SLV: single-locus variant
SQ: South Queensland
ST: Sequence type
